# Invasion of Chicken Anemia Virus in Specific-Pathogen-Free Chicken Flocks and Its Successful Elimination from the Colony

**DOI:** 10.3390/vetsci11070329

**Published:** 2024-07-22

**Authors:** Akira Fujiwara, Wataru Horii, Junichi Sano, Toshiaki Kodama, Atsushi Kato, Kazumoto Shibuya, Toshiki Saitoh

**Affiliations:** 1 Nippon Institute for Biological Science, 9-2221-1 Shin-machi, Ome 198-0024, Tokyo, Japan; fujiwara@nibs.or.jp (A.F.);; 2 Nisseiken Co., Ltd., Kobuchisawa Facility, Kamisasao, Hokuto 408-0041, Yamanashi, Japan; horii@nibs.or.jp (W.H.); sano@nibs.or.jp (J.S.); 3 Biomedical Science Association, 2-20-8-3F Kamiosaki, Shinagawa-ku 141-0021, Tokyo, Japan; 4 Nisseiken Co., Ltd., 9-2221-1 Shin-machi, Ome 198-0024, Tokyo, Japan; t-saitoh@nibs.or.jp

**Keywords:** artificial insemination, chicken anemia virus (CAV), genome sequence, elimination, SPF

## Abstract

**Simple Summary:**

Outbreaks of chicken anemia virus (CAV) had occurred in specific-pathogen-free (SPF) chicken flocks. Removing seropositive chickens and adjacent seronegative chickens from the SPF facility and disinfecting housing with chemical agents did not halt outbreaks. Incidences decreased with an increase in chicks hatched from CAV-seropositive hens as maternal antibodies protected these chicks from CAV infection. We isolated eggs from CAV-seropositive hens through artificial insemination using CAV-free semen from roosters. The eggs were transferred to a new SPF facility and used to produce CAV-free progeny. To date, the colony raised at the new facility has been CAV-free for longer than two years.

**Abstract:**

A specific-pathogen-free (SPF) chicken colony was maintained with successive groups a month apart in age. The absence of specific pathogens, including chicken anemia virus (CAV), was confirmed through periodic serological tests for each group. However, some groups became CAV seropositive. The procedures of removing seropositive and the adjacent seronegative chickens followed with chemically disinfecting the housing did not halt CAV outbreaks. The full genome sequence of the CAV strain that appeared was closely related to low-virulence isolates in China. The outbreaks of CAV decreased with an increase in the seropositive chicken population, indicating that the progeny is protected from CAV infection by maternal anti-CAV antibodies. The persistence of CAV in erythroid and lymphoid tissues or reproductive tissues from CAV seropositive chickens was examined in chickens of various ages using polymerase chain reaction (PCR). Since a low persistence of CAV was observed in the colony, we isolated eggs from CAV seropositive hens through artificial insemination using semen collected from roosters and confirmed as CAV-free by PCR. Fertilized eggs were transferred to a new SPF facility and used for generating CAV-free progeny. To date, chickens reared in the new facility have been CAV-free for longer than two years. Redirection of eggs from seropositive hens was an effective means of eliminating CAV from chickens.

## 1. Introduction

Chicken infectious anemia (CIA) caused by the chicken anemia virus (CAV) is an immunosuppressive disease that results in great economic loss to the poultry industry [1,2,3,4,5]. CAV was first identified as the chicken anemia agent and isolated in 1979 in Japan from diseased chickens with Marek’s disease vaccination failures (reviewed in [6,7]). Since then, it has been renamed as CAV based on virological evidence and has been detected in almost all countries with significant poultry production.

CAV has a single-stranded circular DNA genome consisting of 2298 or 2319 bases with negative polarity and is characterized by a small non-enveloped icosahedral structure with a diameter of 23 to 25 nm [8]. CAV is classified into the *Gyrovirus* genus in the family *Anelloviridae* [9]. A single mRNA is transcribed from the CAV genome having three open reading frames (ORFs), 1, 2, and 3, in a partially overlapping manner. ORFs encode three viral proteins, VP1 (51 kDa), VP2 (24 kDa) and VP3 (14 kDa), respectively [10]. VP1 is the sole structural protein of the CAV virion and is known to induce neutralizing antibodies. VP2 and VP3 are non-structural proteins. VP2 is thought to act as a scaffolding protein to facilitate the correct assembly of VP1 [11]. VP3 is known to induce cell apoptosis in chicken thymocytes and lymphoblastoid T cell lines in vitro [12] as well as in other animal cell lines [13]. VP3 is thought to be associated with the pathogenesis of CIA. Divergence of the CAV genome is observed, although it is around 5% at most [14]. Several classifications such as genogroups I to V [1], genotypes A to C [15], or clades GI and GII [16] have been proposed till now. Since no difference in the antigenicity of CAV isolates has been detected when using chicken antisera, CAV is thought to consist of a single serotype [7].

The disease primarily affects chicks less than 2 weeks old [17], while older chickens more than 3 weeks generally experience subclinical infections [18]. After entering the susceptible chicken body, CAV infects several bone marrow-derived cells such as erythroid and lymphoid precursor, leading to the depletion of these cells and thus resulting in severe anemia and immunosuppression [19]. Usually T-lymphocytes in the thymus and the spleen are severely affected by the infection, while B-lymphocytes are relatively less affected. CAV is transmitted both horizontally and vertically [20]. As a large amount of CAV is excreted in the feces of infected chickens, contaminated feces are the main source of horizontal infection via the oral route. Horizontal CAV transmission via the mucosal route (eyes and nose) is also important because CAV is produced in feather follicle epithelia of infected chickens. Experimental infection of epithelial homogenates taken from an infected chicken caused CAV infection in day-old chicks [21]. Anti-CAV antibodies in hens are transferred to their progeny via egg yolk as maternal antibodies, which protect the offspring from the onset of CIA [18]. Horizontal transmission usually occurs at two weeks or older, which is when maternal antibodies begin to disappear. Horizontal infection normally causes asymptomatic disease accompanied by immunosuppression or leads to secondary infections resulting in economic losses [22]. Vertical transmission occurs in naive progeny through eggs and causes symptomatic disease including anemia, paleness, depression, reduced weight gain, and death (generally 10–20%, but as much as 60% in some cases) in young chickens. This usually occurs when anti-CAV antibody-negative hens become infected with CAV during sexual maturation and then produce eggs [23]. Infected roosters shedding CAV in semen are also a source of vertical transmission. Further, CAV might persist in the reproductive organs of chickens and be passed to the progeny embryo in the hen independently of the presence of anti-CAV antibodies [24,25].

The specific-pathogen-free (SPF) chicken, Line-M, was established in 1969 at the Laboratory Animal Research Station of the Nippon Institute for Biological Science (NIBS) in Yamanashi prefecture in Japan. The Kobuchisawa facility was subsequently donated to Nisseiken Co., Ltd., a partner company of NIBS, in 2018 during an organizational change. These SPF flocks were bred in groups classified according to hatch days and groups were routinely monitored using periodic serological tests of blood taken from randomly selected 60-day-old and 6-month-old chickens to confirm that they were free of CAV infection. However, these flocks became seropositive to CAV despite the strict group-based control. Since CAV is remarkably resistant to chemicals, it is generally thought that the elimination of CAV from contaminated facilities is very difficult [26]. This study discusses efforts to eliminate CAV from chicken colonies following the invasion of CAV in the chicken flocks. We isolated CAV-free eggs from the CAV seropositive hens by employing artificial insemination using CAV-free semen prepared from roosters. Finally, we successfully re-established the SPF chicken colony free from CAV. To date, the CAV-free status in this facility has been maintained for longer than two years. Redirection of eggs from seropositive hens was proven to be effective for eliminating CAV from chicken flocks.

## 2. Materials and Methods

### 2.1. Animal Handling and Breeding

All animal research, including handling and breeding for maintaining the specific-pathogen-free (SPF) chicken colony, was carried out according to the regulations and guidelines established by the Nippon Institute of Biological Science (NIBS) Animal Care and Use Committee. All animal procedures were performed under the principles outlined in the Japanese Animal Welfare and Management Act.

### 2.2. Chickens

Specifications required for SPF chicken frocks are determined by the Japanese regulation (see, Supplementary Materials Table S1). SPF chickens are maintained with filtered air under positive pressure at the Kobuchisawa facility of Nisseiken Co., Ltd. The facility for line-M chickens includes four rooms, an egg hatching room, a chick brooder room, a growing chicken room, and a full-grown chicken room, with a shower-in room for workers and a feedstock room adjacent to the disinfection room. The egg washing room and egg stock room are also attached to the facility. The chicken rooms are kept at 16–28 °C under controlled light (14 h) and dark (10 h) cycles. The chickens are supplied with a gamma ray sterilized formula feed and sterile water for drinking. Workers routinely shower immediately before entering the chicken rooms.

### 2.3. Chicken Semen and Artificial Insemination

The copulatory organ of the rooster was stimulated by stroking the abdomen and back region towards the tail. Then the region around the sides of the cloaca was squeezed gently to get semen from the ducts of the copulatory organ into a small tube. Care was taken to prevent contamination of the semen with cloacal products. The collection procedure was repeated once more, and the semen samples (0.5 to 1.0 mL) were pooled by a rooster. Semen was mixed with the same volume of a solution containing 2 mg/mL glucose, 38 mg/mL trehalose dihydrate, 12 mg/mL sodium hydrogen monohydrate, 3 mg/mL potassium acetate, 0.8 mg/mL magnesium acetate tetrahydrate, 0.5 mg/mL tri-potassium citrate, and 0.01 mg/mL gentamicin and kept at 5 °C for 20 to 30 min. The mixture was then diluted with the same volume of a second solution containing 15% (*v*/*v*) methyl acetamide in the first solution. Then the semen mixture was stored in liquid nitrogen until use. Sperm motility in the thawed semen mixture was checked at intervals before implementing artificial insemination. Portions of semen obtained from CAV-seropositive roosters were checked using a CAV detection PCR and used after they proved to be CAV negative since there were cases of semen contaminated with CAV [24]. Artificial insemination was carried out fundamentally according to an established procedure [27]. Briefly, the left side of the hen’s abdomen around the vent was pushed to cause opening of the vaginal orifice. An inseminator containing the semen was inserted 2.5 cm deep into this opening. Then the semen was expelled from the inseminator. Artificial insemination was usually carried out when hens did not have an egg in the oviduct.

### 2.4. Serological Test for Anti-CAV Antibodies

Sera were prepared from the chicken blood using a standard procedure and were used immediately or otherwise stored at 4 °C until use. Anti-CAV antibody titers were determined using a commercially available anti-CAV antibody ELISA kit (IDEXX Chicken Anemia Virus Antibody Test Kit, IDEXX Laboratories, Westbrook, ME, USA) according to the manufacturer’s recommendations. Briefly, heat-inactivated sera were diluted ten-fold (1/10) with the sample diluent included in the kit and were used for the ELISA. Then, ELISA OD values at 650 nm were calibrated with those obtained from positive and negative sera included in the ELISA kit to obtain a quantitative result for anti-CAV antibodies. Signal-to-noise ratio (S/N) values calculated according to the manufacturer’s instructions were used to determine if samples were CAV-seropositive (S/N ≦ 0.6) or seronegative (0.6 < S/N).

### 2.5. Nucleic Acid Extraction

Organ samples, such as thymus, liver, spleen, bursa of Fabricius, bone marrow, eggshell membrane, semen, and ovary/testis were temporarily stored at −80 °C for subsequent CAV DNA isolation. The frozen organ samples were thawed immediately before homogenization. Chorio-allantoic cavity fluids and blood were used without freezing. Viral DNA was extracted using the DNeasy Qiagen Blood and Tissue kit (Qiagen K.K., Tokyo, Japan) according to the procedure indicated by the manufacturer. The quantity of purified DNA solution was measured using a spectrophotometer NANO DROP LITE (Thermo Fisher Scientific Inc., Tokyo, Japan). All DNA samples were adjusted to concentrations of 10 ng/μL.

### 2.6. CAV Detection by PCR Assays and Full Genome Sequence of CAV

A conventional PCR (cPCR) assay was performed to detect the CAV genome using primer pairs according to the method described by Todd et al. [28]. A 675 bp fragment corresponding to a portion of the *VP1* gene was amplified using the Prime STAR HS DNA polymerase (Takara Bio Inc., Kusatsu, Japan). The lengths of amplified DNA fragments were confirmed using 1.5% (*w*/*v*) agarose gel electrophoresis in Tris–Borate–EDTA (TBE) buffer. If necessary, an amplified fragment was purified using the PCR cleaning kit (NucleoSpin Gel and PCR Clean-up Kit, Macherey-Nagel-Takara Bio Inc., Kusatsu, Japan) and was sequenced by ordering the sequencing service provider (Fasmac Co., Ltd., Atsugi, Japan). In addition, a quantitative PCR (qPCR) was performed to confirm the CAV genome using primer pairs and performed according to the method by Vagnozzi et al. [29] except that the probe was labeled with 5′-FAM and 3′-BHQ1 and that PrimeTime Gene Expression Master Mix reagent (Integrated DNA Technologies Co., Ltd., Tokyo, Japan) was used for the amplification by the StepOnePlus real-time thermal cycler (Thermo Fisher Scientific Inc., Tokyo, Japan). A 181 bp fragment corresponding to the *VP2* and *VP3* overlapping gene region was amplified by qPCR. A 1944 bp DNA fragment of CAV (covering nucleotide positions from 359 to 2302 of the genome) was quantitated using by OD260 of the spectrophotometer NANO DROP LITE and used as the copy number standards (10^2^, 10^3^, 10^4^, 10^5^, 10^6^, and 10^7^ copies/μL). The full genome sequence of the CAV that appeared in the SPF chicken flock was determined by primer walking. The CAV from which the genome was sequenced was named the U4LM5 strain and registered in the DNA database (GenBank accession number: LC817950).

### 2.7. Sequence Alignment and Phylogenetic Analysis of CAV

The full genome nucleotide sequences of the CAV U4LM5 strain were compared with full genome sequences of 48 relevant CAV strains collected from GenBank. Sequence alignment and phylogenetic analysis were performed using the muscle and the maximum likelihood method, respectively, using the MEGA11 free software (megasoftware). The number of nucleotide substitutions was inferred using the Tamura–Nei model by applying Neighbor-Join and BioNJ algorithms with 1000 bootstrap replications. Deduced amino acid sequences of the VP1 protein were also used for comparison.

### 2.8. Identifying the CAV Genome in the Chicken Tissues and the Embryonating Eggs

To search for the CAV genome in chickens, different aged (20-day-old, 30-day-old, 10-month-old, and 12-month-old) chickens were selected for investigation. Discrimination of rooster and hen for these chickens was carried out by the shape of cockscomb. Twenty 20-day-old chicks (10 roosters and 10 hens), six 30-day-old chickens (3 roosters and 3 hens), ten 10-month-old chickens (5 roosters and 5 hens), and ten 12-month-old chickens (5 roosters and 5 hens) bred from the eggs of CAV-seropositive laying hens were sacrificed to survey whether CAV was present or absent in erythroid and lymphoid tissues or the reproductive tissues. Thymus, liver, spleen, bursa of Fabricius, femoral bone marrow, mucosal membrane of the cloaca, and blood were taken from the chickens. The ovary, infundibulum of the oviduct, and isthmus of the oviduct were taken from hens. The testis and epididymis were taken from roosters. Some reproductive tissues such as the infundibulum of the oviduct, isthmus of the oviduct, and epididymis were not collected from sexually immature chicks. The eggshell membrane, chorio-allantoic cavity fluids, and germ spleen of the embryonating eggs were also used for screening following methods described previously [30].

## 3. Results

### 3.1. SPF Chicken Colony

The SPF closed chicken colony called line-M was correctly maintained in NIBS since 1969 and was succeeded by Nisseiken Co., Ltd. The SPF chickens have greatly contributed to health sciences by providing embryonated eggs, chicks, and chickens. Colony groups were classified according to hatching day. A hen group composed of 80 to 100 individuals was produced once a month and was distinguished by naming in alphabetical order. A series of hen groups with a difference of one month in age between successive groups were thus bred in the SPF facility. The quality of the chicken group as an SPF was confirmed serologically and microbiologically when chicken groups reached 8 weeks and 6 months by testing the peripheral blood taken from, respectively, 20 and 30 randomly selected chickens per group. In the case of the serological test of the 6-month-old chicken group, each serum was tested individually, while those of the 8-week-old chicken group were performed for two pooled sera, which were a mixture of sera from 10 individuals.

### 3.2. CAV Invasion in Flocks of SPF Chicken Colony

**Figure 1:**
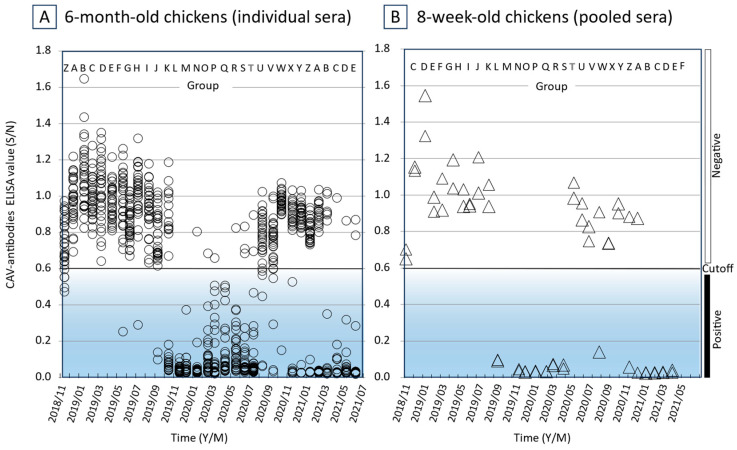
Anti-CAV serum titers in chicken flocks of a CAV-contaminated facility. The vertical axis indicates CAV-antibody ELISA titers (S/N values). The horizontal axis indicates the time (Year/Month) when the ELISA test was run. Hen groups composed of 80 to 100 individuals produced once a month are shown in alphabet (A to Z). (**A**) Thirty sera randomly collected from the 6-month-old chicken groups are shown individually by open circles. (**B**) Twenty sera were collected from 8-week-old chicken groups and data of the sera pooled from 10 individuals are shown by open triangles. The cutoff value is 0.6. Negative and positive regions are shown by the open and closed boxes, respectively.

**Figure 2:**
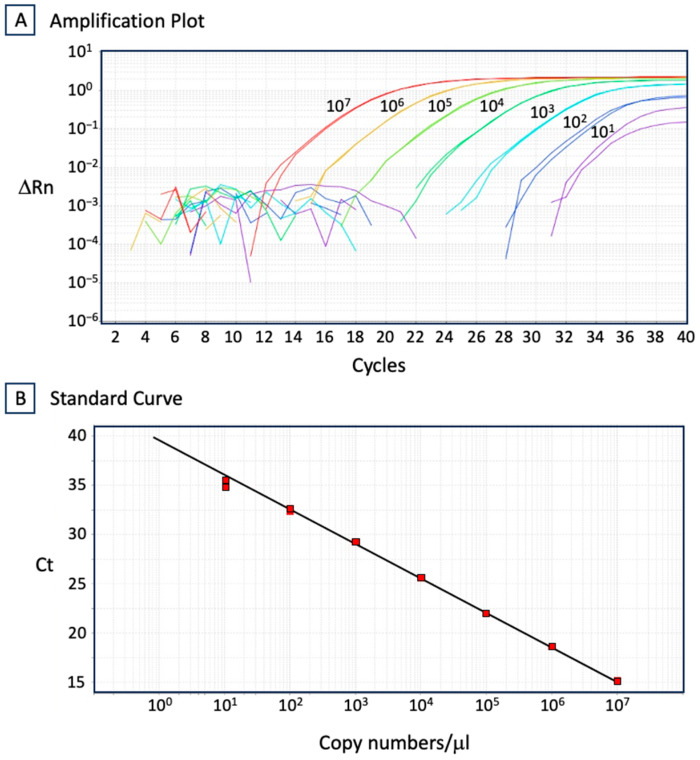
Quantitative PCR detecting the CAV genome. Quantitative PCR (qPCR) was performed as described in Materials and Methods. A 1944 bp DNA fragment of CAV (covering nucleotide positions from 359 to 2302 of the U4LM5 strain) was used as the copy number standards (10^2^, 10^3^, 10^4^, 10^5^, 10^6^, and 10^7^ copies/μL). An example of qPCR is representatively shown. Each reaction for the standards was conducted in duplicate, but that for dose-unknown samples was performed in triplicate. Rn is the fluorescence signal value normalized with ROX Dye (Applied Biosystems/Thermo Fisher Scientific Inc.), and ΔRn is obtained by Rn minus the baseline value. Amplified curves of each copy number standard are shown in different colors in (**A**). Ct (cycle threshold) values of each copy number standard were calibrated by StepOne Plus based on the threshold line. A correlation curve between Ct value and copy numbers is plotted (R^2^ = 0.999, amplification efficiency = 93.128%) in (**B**). Using the correlation curve plotted like this representative, a copy number of the unknown samples was calculated every time together with the copy number standards.

We detected anti-CAV antibodies in 6 out of 30 chickens randomly selected from the 6-month-old Z group tested in November 2018 (Figure 1A), whilst chicken sera collected at the same time from the 8-week-old C group were seronegative for CAV (Figure 1B). The six seropositive chickens and adjacent seronegative chickens (including from both sides, above, below, and behind) were immediately removed from the facility. Moreover, the sites from which chicken cages were removed were disinfected using a sodium hypochlorite solution, and the cages were disinfected with boiling water. The next month’s tests of the 8-week-old D group chickens and the 6-month-old A group chickens performed in December 2019 were both seronegative to CAV. Seronegative conditions continued for the 6-month-old chicken tests in several successive groups (A to E), but again 1 out of 30 chickens randomly selected from the F group was CAV-seropositive when the tests were performed in May 2019 (Figure 1A). Again, this was followed by the immediate removal of the seropositive chicken and adjacent chickens. The next tests of the 6-month-old G group chickens performed in June 2019 were seronegative, but in the H group tests performed in July 2019, 1 out of 30 chickens was once again CAV-seropositive. From the initial occurrence of CAV in the 6-month-old chickens in November 2018 until October 2019, the 8-week-old chicken groups (C to L) were kept seronegative to CAV, but eventually became seropositive (Figure 1B). Moreover, of the 6-month-old L group chickens tested in December 2019, all 30 randomly selected chickens were seropositive to CAV. These results indicated that CAV was not eliminated from the facility by simply removing the seropositive chickens and adjacent seronegative chickens and by follow-up with disinfection procedures. The CAV genome was detected from the blood samples of CAV ELISA-positive chickens using both cPCR and qPCR. The example quantity of the CAV genome in the blood (No. 4 of the U group) was 217.2 ± 22.1 copies/μL according to our qPCR procedure (Figure 2).

### 3.3. Full Genome Sequence of CAV Appeared in the Chicken Flock

**Figure 3:**
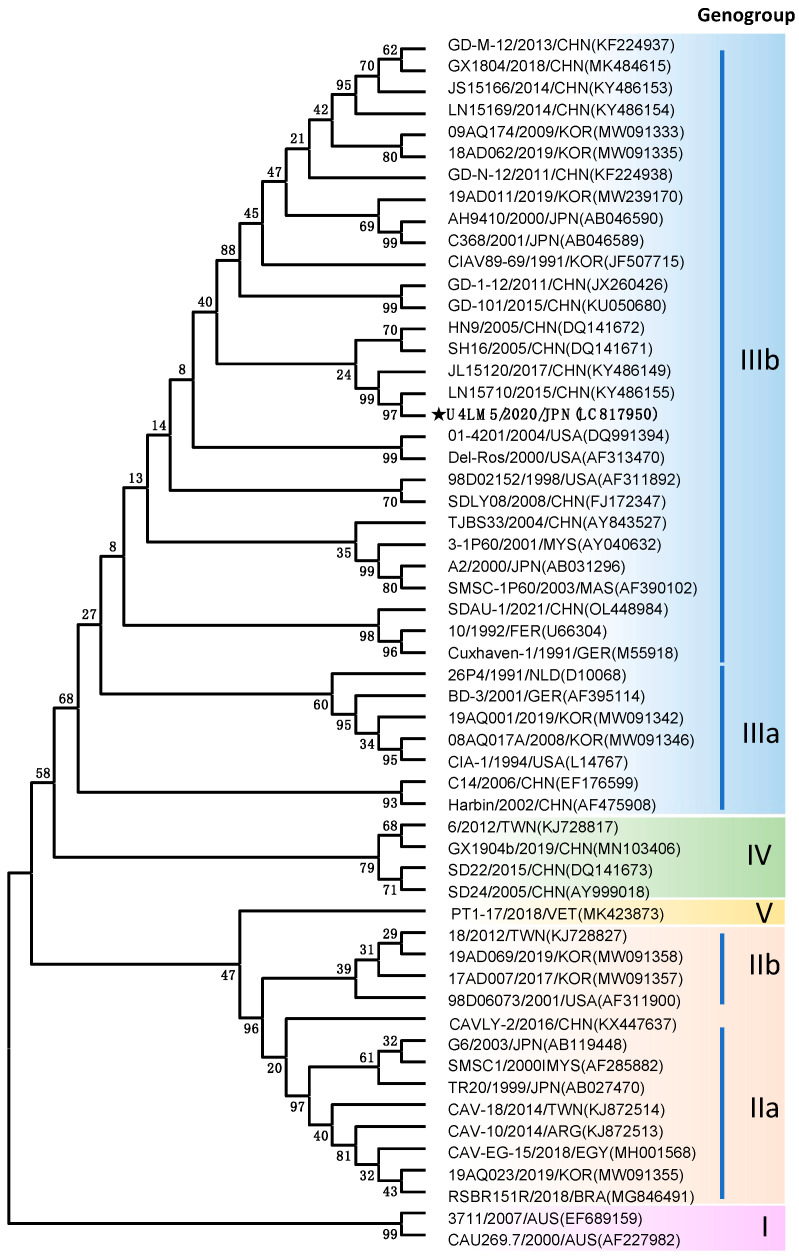
Phylogenic tree of CAV U4LM5 obtained from an SPF chicken in the facility. A phylogenic tree based on the whole genome sequence of CAV is shown. The CAV U4LM5 strain is shown in bold with a star. Relevant full genome sequences of CAV strains collected from GenBank are shown with the DNA database accession number. Five genogroups, I to V, of CAV strains classified using the Maximum Likelihood method are shown. Sub-genogroups (IIa, IIb, IIIa, and IIIb) further classified from genogroups II and III are also shown. The bootstrap values obtained from 1000 replicates of the bootstrap test are indicated at each branch node of the tree. Evolutionary analyses were conducted in MEGA11 software.

Although the appearance of CAV in the chicken flocks was confirmed using anti-CAV antibody ELISA and PCR, noticeable symptoms of CAV-positive chickens were not observed. This silent invasion of CAV made it difficult to understand the full extent of the CAV contamination in the facility. Thus, we decided to provisionally stop the supply of SPF chicken and to maintain the facility as CAV contaminated. The full genome sequence of the CAV U4LM5 [GenBank accession number: LC817950] isolate obtained from chicken No. 4 of the U group was identified using a primer working method. The genome of the CAV U4LM5 strain is composed of 2298 bases and is classified into the genogroup IIIb (Figure 3). Several amino acid substitutions found in the VP1 protein are related to viral pathogenicity and growth ability (Table 1). At amino acid positions 75 and 128 of the VP1 protein, those which might be associated with lower pathogenicity are valine (V) and leucine (L) in the U4LM5 strain, respectively. Methionine (M) at position 157, which is unique to the low pathogenic strain of genogroup IIIb CAV, is found in the U4LM5 strain. The pattern of amino acids at positions 139, 144, and 287, which is predicted to be associated with viral growth in the cells, is lysine (K), glutamic acid (E), and threonine (T) in the U4LM5 strain, respectively.

The deduced amino acid sequences of VP1 proteins of known genogroups are compared with the CAV U4LM5 strain. Amino acid changes at specific positions are shown by a single letter code.

### 3.4. Anti-CAV Antibodies in the Chicken Groups

Interestingly, the number of CAV seronegative chickens in the facility increased after the 8-week-old U group chickens were tested in May 2020 (Figure 1B). This was also observed after the 6-month-old T group chickens were tested in August 2020 (Figure 1A). The situation with the majority of chickens being CAV seronegative continued for several months until November 2020, when the 8-week-old Z group chickens were tested and March 2021, when the 6-month-old B group chickens were tested. The change from seropositive to seronegative may be explained by protection from the CAV infection obtained from maternal antibodies which chicks received when they were highly susceptible to the CAV. However, as the number of CAV-antibody-negative hens again increased, by escaping the CAV infection at a young age, it was thought that CAV-susceptible chicks without maternal antibodies against CAV were hatched from those hens and that these hatched chicks were infected with CAV and became antibody-positive.

### 3.5. Age-Dependent Changes of Anti-CAV Antibodies Titer

**Figure 4:**
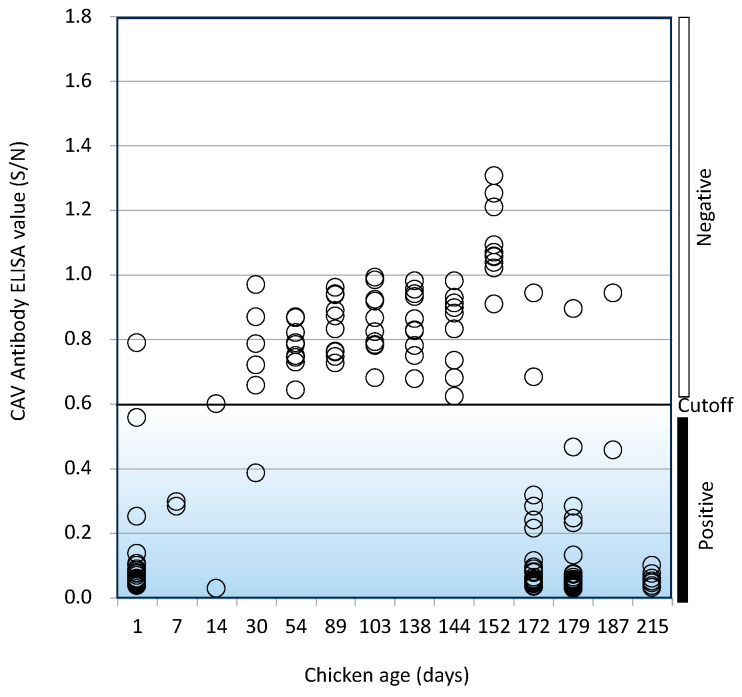
Age-dependent anti-CAV serum titers in a CAV contaminated facility. The vertical axis indicates CAV-antibody ELISA titers (S/N). The horizontal axis indicates the age (in days) of chicks or chickens from which serum was taken. Each ELISA titer is shown by an open circle. The cutoff value is 0.6. Negative and positive regions are shown by the open and closed boxes, respectively.

To understand the age-dependent dynamics of anti-CAV antibodies in chicks and chickens, we randomly collected sera from chicks and chickens of different ages (from 1 to 215 days) during a period when the 6-month-old chicken test results were CAV seropositive and measured the anti-CAV antibodies ELISA titer (Figure 4). Except for one chick, one-day-old chicks had anti-CAV maternal antibodies. The maternal antibodies could be detected until 15 days after hatching but were barely detectable 30 days after hatching. This indicated that anti-CAV maternal antibodies could protect against CAV infection post hatching (less than 2 weeks old), a stage when chicks are highly susceptible to CAV. Anti-CAV antibodies were negative in chickens from 30 to 152 days old but became positive in chickens from 172 days and older. This re-appearance of CAV-antibodies was thought to either indicate horizontal infection of CAV after the disappearance of maternal antibodies in the facility, or alternatively to indicate re-activation of CAV which had infected chickens during the first two weeks after hatching and had persisted in the body, without causing apparent illness, but appeared during sexual maturation.

### 3.6. Persistence Infection of CAV in Lymphoid, Hematopoietic, or Reproductive Tissues

To increase understanding of the dynamics of CAV infection, we searched for the CAV genome in several organs and blood (Table 2). Twenty 20-day-old chicks, which had maintained the maternal antibodies to CAV, and six 30-day-old chicks, which had lost the maternal antibodies to CAV, were sacrificed and CAV-sensitive lymphoid or hematopoietic tissues (spleen, bursa of Fabricius, thymus, liver, and bone marrow), reproductive tissues (testes from roosters, ovaries and infundibula of the oviduct from hens), and blood were examined using CAV-specific PCRs (Table 2). The PCR sensitivity was confirmed to be more than 10 copies per 10 ng of DNA using the positive control in every PCR tests. CAV genomes were not detected in any of these tissues, suggesting that these chicks were protected from the CAV infection by maternal antibodies and that non-apparent infection of CAV in these chickens was unlikely. However, the number of chickens used for this study was insufficient to be conclusive. We next examined ten 10-month-old and ten 12-month-old hens and roosters which were seropositive for CAV to identify whether the CAV infection leading to the seroconversion had occurred through invasion of external CAV that came from outside of the chicken body or through the reactivation of internal CAV that had remained in the chicken body. The CAV genome was not detected in the lymphoid or the hematopoietic tissues (spleen, bursa of Fabricius, bone marrow, and liver), or the blood of 10-month-old chickens. However, the CAV genome was detected in the ducts deferens of two of the five 10-month-old roosters (Table 2). In the two CAV-positive samples, the qPCR procedure established that the CAV genome was 55.0 ± 17.0 and 10.8 ± 13.7 copies/μL (Figure 2). Although the latter was below the detection limit of the qPCR (>10 copies/μL) considering the deviation, both were also detected through conventional PCR (see, Supplementary Materials Figure S1). Interestingly, the CAV genome was not detected in the testes of the five roosters or the reproductive tissues (ovary, infundibulum of the oviduct, and isthmus of the oviduct) of the five hens. These results indicated that CAV occurs infrequently in reproductive tissues and were limited. Further, the CAV-seropositive 12-month-old chickens were examined to establish the presence of the CAV genome, but it was not detected in the testes or reproductive tissues of any of the roosters and hens. This result supports the notion that the persistence of CAV in reproductive tissues occurs infrequently. However, the CAV genome was detected in the thymus of one of the five hens. No CAV genome was found in other lymphoid or hematopoietic tissues (spleen, bursa of Fabricius, bone marrow, and liver), or in blood. The CAV genome in the thymus was quantitated using qPCR as 266.1 ± 6.7 copies/μL. We reasoned that the presence and absence of CAV in lymphoid or hematopoietic tissues of CAV-seropositive chickens were probably related to the time lapse since infection with external CAV and not due to reactivation of persistently infected internal CAV.

### 3.7. Re-Establishment of SPF Chicken Colony

**Figure 5:**
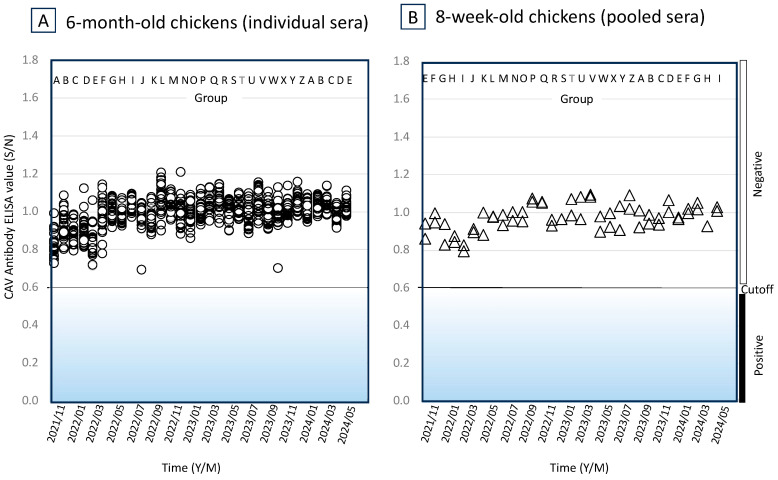
Anti-CAV serum titers in chicken flocks of a CAV-eliminated facility. The vertical axis indicates CAV-antibody ELISA titers (S/N). The horizontal axis indicates the time (Year/Month) the ELISA test was run. Hen groups composed of 80 to 100 individuals produced once a month are shown in alphabet (A to Z). (**A**) Thirty sera randomly taken from 6-month-old chicken groups are shown individually by open circles. (**B**) Twenty sera collected from 8-week-old chicken groups were pooled into two samples of 10 individuals and both pooled samples are shown by two open triangles for each date. The cutoff value is 0.6. Negative and positive regions are shown by the open and closed boxes, respectively.

Given that chicks hatching from eggs of CAV-seropositive hens were protected from CAV infection and were kept CAV-free for a while in the facility, we attempted to re-establish the SPF chicken colony using the embryonating eggs obtained from CAV-seropositive hens. Thus, we built a new facility for the SPF chicken colony and stipulated stricter operational procedures and regulations for husbandry of SPF chickens. For example, we formerly relied upon natural copulation between hens and roosters, but in the new facility we introduced the use of artificial insemination using rooster semen that was pre-qualified by PCR as CAV-free and stocked in liquid nitrogen. Almost one hundred embryonating eggs were obtained from CAV-seropositive D group hens whose peripheral bloods were pre-qualified by PCR as CAV-free. Eggs were also analyzed using PCR to determine if CAV was in the eggs. Eggshell membrane, chorio-allantoic cavity fluids, and germ spleen of the embryonating eggs were PCR screened for CAV, but CAV was not detected in any egg samples. Eggs obtained from the same hens on different days were then chemically disinfected and moved to the new facility. Overall, 102 chicks were hatched from the eggs in the new facility and were classified as A group of the F1 generation. At 30 days old, roosters were thinned out from the A group, but a few were kept as a source of semen, and their blood was PCR screened. Sera of 52 hens were checked at 32-, 49-, 60-, and 80-days-old using anti-CAV antibody ELISA, and blood obtained from 49-day-old chickens were PCR screened Results indicated that they were CAV-negative. The F1 eggs which were obtained from CAV seronegative A group hens by artificial insemination using the CAV-free semen were incubated to produce the B group of the F2 generation. Likewise, production of successive groups was continued in the new facility. The chicken colony in the old facility contaminated with CAV was terminated as the new facility started functioning. The old facility was disinfected extensively to remove any contamination source for the new facility. The SPF specification test for CAV of a 6-month-old (Figure 5A) and an 8-week-old (Figure 5B) chicken clearly indicated that the CAV-free status of the chicken colony was maintained from October 2021 to May 2024. This result indicated the successful elimination of CAV from the SPF colony and the re-establishment of the line M SPF colony.

## 4. Discussion

SPF animals are usually maintained under strictly controlled clean conditions. However, contamination or susceptible cases do uncommonly occur, even in the SPF facility [31,32]. CAV infections in SPF chickens were reported at Cornell University, NY [33]. A CAV outbreak in flocks in an SPF chicken breeding facility also occurred in the Kobuchisawa facility of Nisseiken Co., Ltd. The CAV infection in chickens appeared without visible symptoms but was detected serologically through routine SPF chicken testing. No clear CAV entry route into the facility could be identified. We immediately eliminated the CAV-seropositive chickens and their adjacent chickens and chemically disinfected the housing and location. However, the CAV infection was not eradicated in the facility. This is consistent with prior research indicating that CAV is strongly resistant to chemical reagents, and it has long-term survival in the facility environment [26,34].

In our institute, two SPF chicken breeding facilities (both for line-M) were in operation at the same time to respond to the demand for SPF chickens without breaks in continuity. This operational procedure was thought to make matters worse because the CAV infection was found in succession in the second chicken breeding facility. We faced a disastrous loss of the SPF chicken colony, which is important in the health science fields, such as for the development of vaccines and their quality control tests. Since the chicken groups passed the SPF grade tests except for CAV, we provisionally continued to operate the chicken breeding facility as a CAV seropositive facility and observed the chicken flocks carefully by monitoring anti-CAV antibodies using a CAV ELISA kit and CAV genome using CAV-specific PCR. Meanwhile, we revised the working procedures in the SPF chicken facility, and decided to build a third facility so as to establish an operation where two of the three facilities were working and one facility was resting as a cleanup period, and the resting facility was rotated periodically.

A genome of the CAV U4LM5 strain (Gene Accession No. LC817950) found in our SPF chicken facility has several characteristics specific to low virulent viruses such as valine (V), leucine (L), and methionine (M) at amino acid positions 75, 128, and 157 of the VP1 protein (Table 1). Highly similar sequences were found in isolates in the LN15710 and JL15120 strains (Gene Accession No. KY486155 and KY486149, respectively) isolated in China (Figure 2). These two strains have the character of a low virulent CAV strain [22,35], which is consistent with our findings for the U4LM5 strain. Our approach was to re-establish the SPF colony by isolating fertilized eggs from CAV-seropositive hens which had been artificially inseminated with CAV-free semen. There are several reports indicating that CAV can survive in lymphoid tissues [36], hematopoietic tissues [3,37,38], and reproductive tissues [24]. However, the CAV U4LM5 strain disappeared from the tissues when the chickens seroconverted to CAV except for a few cases. Another report indicated that the CAV gene could be detected in the organs of progenies from chickens with a high tier of anti-CAV neutralizing antibodies [25]. However, no CAV was detected in the samples taken from a hundred A group F1 progenies hatched from chickens with the high tier of anti-CAV ELISA serum. To date, no anti-CAV serum has been detected in the series of successive SPF chicken flocks during the routine testing of 8-week-old and 6-month-old chickens (Figure 4). These results indicated the success of the re-establishment of the SPF chicken colony. The success of our strategy may have been aided by the low virulence of the CAV U4LM5 strain. Thus, it should be noted that our strategy does not guarantee the elimination of CAV from a chicken flock in all situations.

## Figures and Tables

**Table 1:** Amino acid variations in the VP1 protein of CAV.

Genogroup	Amino Acids at Specific Positions of the VP1 Protein												
	22	75	97	125	139	144	157	287	290	294	370	376	413
I	H	V	M	I	K	E	V	S/T	A	Q	G/R	L	S
IIa	H	I	L	I	Q	Q	V	T	A/P	H/Q	S	L	A
IIb	Q	I	L	I	Q	Q	V	T	A	Q	S	L	A
IIIa	Q/N	V	L	I	Q	Q	V	A	A	Q	S	L	A
IIIb	H/N/Q	V	L/M	I/L	K	E/Q	M/V	A/S/T	A/P	H/Q	G/S	I/L	A/S
IV	H	V	M	L	K	E	V	A/S	A	Q	S	L	A
V	Q	I	L	I	Q	Q	V	S	A	Q	G	I	A
Isolate													
U4LM5	H	V	L	L	K	E	M	T	P	H	A	L	A

**Table 2:** Occurrence of the CAV genome in organs and blood of different aged chickens.

Chickens		Spleen	Bursa of Fabricius	Thymus	Liver	Femoral Bone Marrow (Left)	Blood	Cloaca, mucous Membrane	Testis	Epididymis (Left)	Ductus Deferens (Left)	Ovary	Oviduct, Infundibulum	Oviduct, Isthmus
20-day-old Q group chicks CAV ELISA Positive	Male	0/10	0/10	0/10	0/10	0/10	0/10	-	0/10	-	-	-	-	-
	Female	0/10	0/10	0/10	0/10	0/10	0/10	-	-	-	-	0/10	-	-
30-day-old Q group chicks CAV ELISA Positive	Male	0/3	0/3	0/3	0/3	0/3	0/3	0/3	0/3	-	-	-	-	-
	Female	0/3	0/3	0/3	0/3	0/3	0/3	0/3	-	-	-	0/3	0/3	-
10-month-old Q group chickensCAV ELISA Positive	Male	0/5	0/5	0/5	0/5	0/5	0/5	0/5	0/5	0/5	2/5	-	-	-
	Female	0/5	0/5	0/5	0/5	0/5	0/5	0/5	-	-	-	0/5	0/5	0/5
12-month-old O group chickensCAV ELISA positive	Male	0/5	0/5	0/5	0/5	0/5	0/5	0/5	0/5	0/5	0/5	-	-	-
	Female	0/5	0/5	1/5	0/5	0/5	0/5	0/5	-	-	-	0/5	0/5	0/5

## Data Availability

The datasets analyzed during the current study are available from the corresponding author upon reasonable request.

## Supplementary Materials

The following supporting information can be downloaded at: https://www.mdpi.com/article/10.3390/vetsci11070329/s1, Table S1: Inspection and Treatment of SPF Chicken Flocks. Figure S1: Conventional PCR (cPCR) for the ducts deferens samples taken from five 10-month-old male chickens.
