# Supplementary material for: Invasion of Chicken Anemia Virus in Specific-Pathogen-Free Chicken Flocks and Its Successful Elimination from the Colony
Source: Vet Sci. 2024 Jul 22;11(7):329. doi: 10.3390/vetsci11070329 (PMC11281415; doi:10.3390/vetsci11070329)
Supplement: Supplementary file 1 [file vetsci-11-00329-s001.zip › Supplemental Table S1.pdf]

# Inspection and Treatment of SPF Chicken Flocks*

| Pathogen | Antigen under Inspection[1] | Inspection timing and number of chickens inspected | | | | Inspection method[2] | Treatment |
|---|---|---|---|---|---|---|---|
| | | 1st | | 2nd and subsequent | | | |
| | | Timing | Number of chickens (%) | Timing | Number of chickens (%) | | |
| Newcastle disease virus | Ishii strain | 8–12 weeks old | 20 | Every 3 months | 10 | HI | All the chickens positive to the inspection and cohabitant chickens[3] sacrificed |
| Avian infectious bronchitis virus | M-41 strain | 〃 | 〃 | 〃 | 〃 | ELISA | 〃 |
| Avian leukosis virus | Sub-A, B | 〃 | 〃 | 〃 | 〃 | SN | 〃 |
| Avian encephalomyelitis virus | Van Roekel strain | 〃 | 〃 | 〃 | 〃 | ELISA | 〃 |
| Avian nephritis virus | G-4260 strain | 〃 | 〃 | 〃 | 〃 | FA | 〃 |
| Infectious laryngotracheitis virus | NS-175 strain | 〃 | 〃 | 〃 | 〃 | ELISA | 〃 |
| Reticuloendotheliosis virus | T strain | 〃 | 〃 | 〃 | 〃 | FA | 〃 |
| Marek's disease virus | JM strain | 〃 | 〃 | 〃 | 〃 | FA | 〃 |
| Infectious bursal disease virus | J1 strain | 〃 | 〃 | 〃 | 〃 | ELISA | 〃 |
| Avian reovirus | Uchida strain | 〃 | 〃 | 〃 | 〃 | DID | 〃 |
| Avian adenovirus | Ote strain | 〃 | 〃 | 〃 | 〃 | DID | 〃 |
| EDS-76 virus | JPA-1 strain | 〃 | 〃 | 〃 | 〃 | HI | 〃 |
| Avian influenza virus | 5331 strain | 〃 | 〃 | 〃 | 〃 | DID | 〃 |
| Chicken anemia virus | Gifu-1 strain | 〃 | 〃 | 〃 | 〃 | FA | 〃 |
| Turkey rhinotracheitis virus | MM-1 strain | 〃 | 〃 | 〃 | 〃 | FA | 〃 |
| Avian paramyxovirus | Yucaipa strain | 〃 | 〃 | 〃 | 〃 | HI | 〃 |
| *Haemophilus paragallinarum* type A | 221 strain | 〃 | 〃 | 〃 | 〃 | HI | 〃 |
| *Haemophilus paragallinarum* type C | S1 strain | 〃 | 〃 | 〃 | 〃 | HI | 〃 |
| *Salmonella pullorum* | 9-25 strain | 〃 | 〃 | 〃 | 〃 | AGG | 〃 |
| *Mycoplasma gallisepticum* | S6 strain | 〃 | 〃 | 〃 | 〃 | AGG | 〃 |
| *Mycoplasma synoviae* | WVU-1853 strain | 〃 | 〃 | 〃 | 〃 | AGG | 〃 |
| Salmonella (except for *Salmonella pullorum*) | - | 〃 | 〃 | 〃 | 〃 | Bacterial isolation | 〃 |
| Chickenpox virus | - | Everyday | 100 | Everyday | 100 | Clinical observation | Chickens positive to the inspection sacrificed |

*This specification is officially notified from the Ministry of Agriculture, Forestry and Fisheries as a part of Minimum Requirements for Veterinary Biological Products.
The official Japanese version is shown on the website (https://www.maff.go.jp/nval/kijyun/seizaikijyun/index.html). An English version was made by the Japan Veterinary Products Association.

**Note**; Health conditions and abnormalities of the chickens shall be completely recorded. For dead chickens, histopathological examinations shall be performed.

1. For antigens under inspection, other appropriate strains may be used.
2. Other similar inspection methods may be used, if any. The inspections shall be performed by a method whose validity has been verified and guaranteed. HI: Hemagglutinating test, ELISA: Enzyme-linked immunosorbent assay, SN: Serum neutralization test, FA: Fluorescence antibody test, DID: Double immunodiffusion test, AGG: Agglutination test
3. Cohabitant chickens refer to the chickens not completely isolated from those positive to the inspection.
