# Supplementary figures and images for: Invasion of Chicken Anemia Virus in Specific-Pathogen-Free Chicken Flocks and Its Successful Elimination from the Colony
Source: Vet Sci. 2024 Jul 22;11(7):329. doi: 10.3390/vetsci11070329 (PMC11281415; doi:10.3390/vetsci11070329)

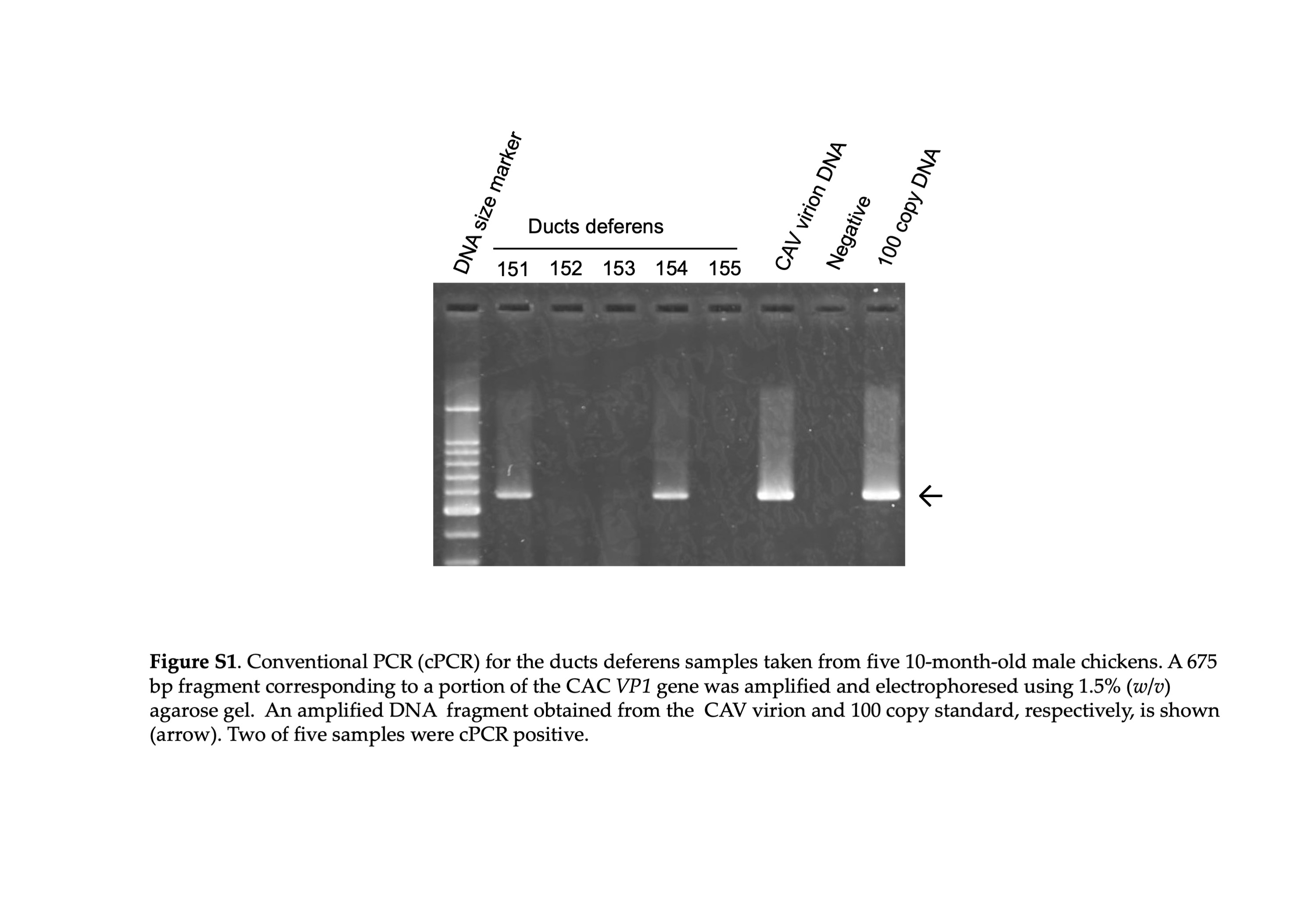

Supplement: Supplementary file 1 [file vetsci-11-00329-s001.zip › Supplemental Figure S1.jpg]
